# Transmission-Blocking Vaccines: From Conceptualization to Realization

**DOI:** 10.4269/ajtmh.22-0023

**Published:** 2022-07-11

**Authors:** Louis H. Miller, Patrick E. Duffy, Richard Culleton

**Affiliations:** ^1^ Laboratory of Malaria and Vector Research, National Institute of Allergy and Infectious Diseases, National Institutes of Health, Bethesda, Maryland;; ^2^ Laboratory of Malaria Immunology and Vaccinology, National Institute of Allergy and Infectious Diseases, National Institutes of Health, Bethesda, Maryland;; ^3^ Division of Molecular Parasitology, Proteo-Science Center, Ehime University, Japan

The complex life cycle of the malaria parasite provides multiple life stages against which to design a vaccine. Vaccines targeting the sporozoite and liver stages can prevent the establishment of a blood-stage infection, those that target the asexual blood stages can prevent disease, and those targeting gametes or ookinetes can prevent transmission by mosquitoes. It is with this latter strategy, that of “transmission-blocking vaccines,” that Richard Carter’s name is most indelibly linked, and forms the subject of this supplement.

The concept of transmission-blocking vaccines and the early work undertaken on them are described in a book by Richard Carter,^1^ which appears in an abbreviated form in this supplement.^2^ Richard performed many studies on the gametocytes of *Plasmodium gallinaceum* and developed a “suspended animation” solution that maintained their viability and allowed vaccination with gametes to produce the first transmission-blocking vaccines. These vaccines induced antibodies that killed gametes and ookinetes in the mosquito midgut. He transferred his work to *Plasmodium falciparum* to identify the surface proteins on gametes—work that was also undertaken by Joep Meuwissen at the University of Nijmegen in The Netherlands. They both used monoclonal antibodies and biochemical methods to identify key molecules on the gamete surface (Pfs230, Pfs48/45, and Pfs47) and the ookinete surface (Pfs25). Each of these proteins is discussed in separate articles in this supplement, and their potential as vaccine targets is evaluated.

Why is this subject so important? Eliminating malaria from Africa is a huge challenge because the malaria vectors on that continent (the *Anopheles gambiae* and *Anopheles funestus* complexes) are remarkably efficient, leading to an R_o_ (R_naught_ is the number of people infected from one infected person) of more than 100 in some regions. When *Anopheles arabiensis* (of the *An. gambiae* complex) was accidently introduced into Brazil, epidemics occurred where none were previously seen. Transmission-blocking vaccines offer the potential to break the cycle of malaria transmission. The goal is to design and produce transmission-blocking vaccines of sufficient efficacy to eliminate *P. falciparum* in such high-transmission settings. A subsequent challenge will be to convince communities to adopt a vaccine that is outwardly altruistic, in that it offers no protection to the individual against the asexual parasites that cause disease. With “vaccine hesitancy” seemingly increasing globally, this may prove a difficult task, although communities impacted by malaria typically express enthusiasm at the prospect of malaria vaccines.^3^

It is likely that a combination of transmission-blocking and pre-erythrocytic vaccines will be required to reduce the prevalence of malaria infection significantly, and a blood-stage vaccine may also be needed to protect children and pregnant women from disease after breakthrough infections.

In this supplement, we focus exclusively on transmission-blocking vaccines and present a series of articles that charts their development from the discovery and characterization of key parasite proteins through to their development as potential vaccines. Pfs230 is the target of the most advanced transmission-blocking vaccine candidates, and the story of its discovery, characterization, and path to clinical development and testing is described by Patrick Duffy,^4^ whose research team at National Institute of Allergy and Infectious Diseases brought the first Pfs230 products into phase I and II clinical trials. Robert Sauerwein and colleagues,^5^ who recently initiated trials with a Pfs48/45 candidate, review the challenges and progress in generating a properly folded Pfs48/45 antigen able to induce functional antibodies suitable for human testing. The discovery and validation of Pfs47 as a transmission-blocking vaccine target, as well as efforts to improve its immunogenicity, is reviewed by Alvaro Molina-Cruz and Carolina Barillas-Mury.^6^ David Kaslow,^7^ who led the efforts that cloned the genes for Pfs230 and Pfs25, summarizes the pioneering work on vaccine candidates based on Pfs25, the first transmission-blocking antigen to undergo testing in humans.

Richard Carter was a pioneer in describing malaria parasite transmission-stage biology, and played a leading role in the development of the concept of transmission-blocking vaccines. His accomplishments in the field range from his description of crucial aspects of gametocyte biology, his realization and proof that antibodies against gametes could block mosquito infection, and his solving of the six-cysteine domain structure of Pfs230. We hope that this supplement serves as a fitting tribute to his work, and that it may inspire a new generation of scientists to pursue this fascinating and crucially important aspect of malariology.
